# Impact of the involvement of hospitals in Benin in the actions of the world program “Save Lives: Clean Your Hands» edition 2014

**DOI:** 10.1186/2047-2994-4-S1-P251

**Published:** 2015-06-16

**Authors:** TA Ahoyo, S Assavedo, AK Aissi, L Toko, TB Orou Yorou, DA Kinde Gazard

**Affiliations:** 1 Point Focal Sécurité Patient/DNSP/Ministère Santé Bénin, Cotonou, Benin; 2 Direction Nationale de la Santé Publique, Point Focal Sécurité Patient/DNSP/Ministère Santé Bénin, Benin; 3 Direction Nationale des Etablissement Hospitalier, Ministère de la Santé, Benin; 4 Ministère de la Santé, Cellule Sécurité des Patients, Benin; 5 Ministère de la Santé, Directeur National Adjointde Santé Publique, Benin; 6 Direction Nationale de la Santé Publique, Benin; 7 Cabinet, Ministère de la Santé, Cotonou, Benin

## Introduction

Benin is engaged in the promotion of the patient safety through the Minister of health. Convinced by the role of hand hygiene in the fight against the spread of pathogens both in hospitals and in the community, the Ministry of Health has encouraged Benin Hospitals which belong to the global initiative “Saves Lives: Clean Hands Yours”.

## Objectives

Improve Patient Safety through the promotion of an intervention-oriented program based on hand hygiene

## Methods

The staff of 40 health facilities (public and private) of Benin was sensitized for two months. Twenty-nine of them accepted to participate in two major WHO surveys in 2014. Data were entered into the http://www.who.int/gpsc/5may/register/fr/site. All hospitals have been associated with the official celebration of the World Day for Hand Hygiene May 5, 2014

## Results

A lack of harmonization and bad evaluation of antibiotic prophylaxis in surgery protocols was found in 55% hospitals. 35% of hospital laboratories have a section dedicated to microbiology. Monitoring of multi-resistant bacteria is insufficiently. The Minister of Health has signed on behalf of the Government the commitment of Benin for the management of care associated infections. Benin was recognized in the 2014 Global-Map published by WHO reflecting the involvement of Benin hospitals in promoting hygiene Hands.

## Conclusion

Benin’s Hospitals should be encouraged by the continuous training of the promotion of correct hand hygiene. The Ministry of Health must be supported to adopt a comprehensive policy and set up a National Patient Safety Program and Risk Management

## Disclosure of interest

None declared.
